# Supplementary material for: Gradient SERS Substrates with Multiple Resonances for Analyte Screening: Fabrication and SERS Applications
Source: Molecules. 2022 Aug 10;27(16):5097. doi: 10.3390/molecules27165097 (PMC9414786; doi:10.3390/molecules27165097)
Supplement: Supplementary file 1 [file molecules-27-05097-s001.zip › molecules-1848382-supplementary.pdf]

Supporting Information

Gradient SERS substrates with multiple resonances for analyte screening: Fabrication and SERS applications

Ashutosh Mukherjee [1,2,3], Quan Liu [3,4], Frank Wackenhut [1,2,*], Fang Dai [5,6], Monika Fleischer [5,6], Pierre-Michel Adam [4,*], Alfred J. Meixner [3,6] and Marc Brecht [1,2,3,6,*]

[1] Center for Process Analysis and Technology (PA&T), School of Applied Chemistry, Reutlingen University, 72762 Reutlingen, Germany; ashutosh.mukherjee@reutlingen-university.de

[2] Reutlingen Research Institute (RRI), Reutlingen University, 72762 Reutlingen, Germany

[3] Institute of Physical and Theoretical Chemistry, Eberhard Karls University of Tübingen, 72076 Tübingen, Germany; quan.liu@uni-tuebingen.de (Q.L.); alfred.meixner@uni-tuebingen.de (A.J.M.)

[4] Laboratory Light, nanomaterials & nanotechnologies–L2n and CNRS EMR 7004, University of Technology of Troyes, 10000 Troyes, France

[5] Institute for Applied Physics, Eberhard Karls University of Tubingen, 72076 Tübingen, Germany; daifang1990@gmail.com (F.D.); monika.fleischer@uni-tuebingen.de (M.F.)

[6] Center for Light-Matter-Interaction, Sensors and Analytics (LISA+), Eberhard Karls University of Tübingen, 72076 Tübingen, Germany

* Correspondence: frank.wackenhut@reutlingen-university.de (F.W.); pierre_michel.adam@utt.fr (P.-M.A.); marc.brecht@reutlingen-university.de (M.B.)

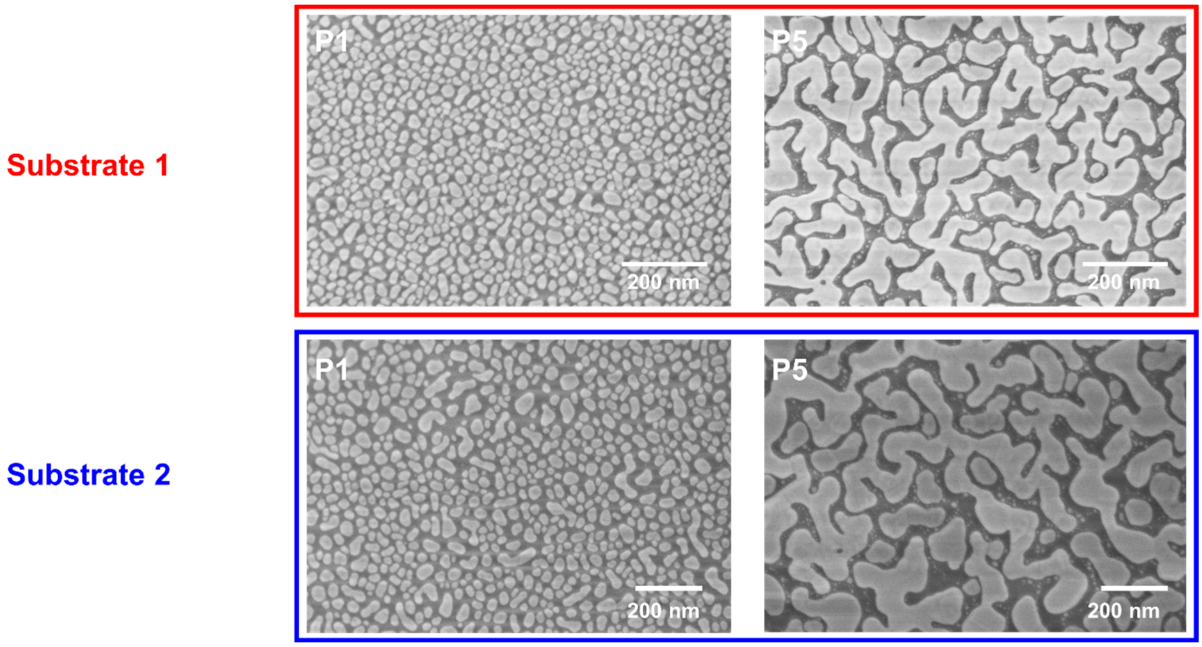

Figure S1. SEM images of different fabricated substrates (substrate 1 - red and substrate 2 - blue) at the same positions P1 and P5 to denote micro- and nano scale reproducibility.

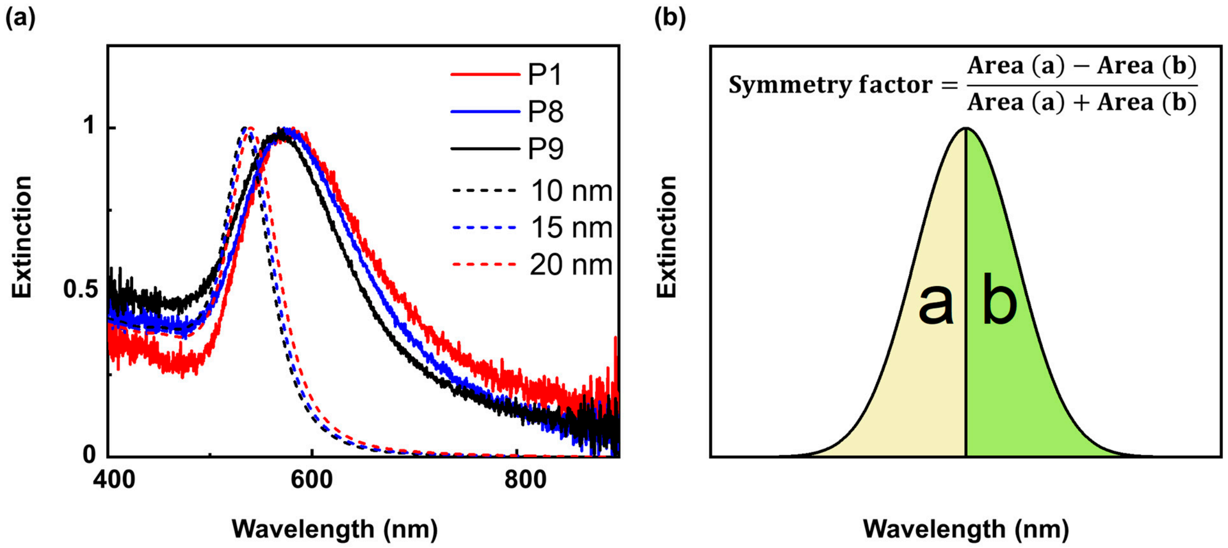

Figure S2. (a) Normalized extinction spectra at points P1, P8, and P9 (solid lines – red, blue, black) compared to normalized simulated extinction spectra from Mie theory for spherical gold NPs with radii of 10 nm, 15 nm, and 20 nm (dashed lines – red, blue, black), and (b) schematic and equation to determine the symmetry factor of an extinction curve.
